# Surgical Burden of Breast Cancer Treatment: Implications of Mastectomy, Breast Conservation, and Reconstruction Choices

**DOI:** 10.3390/medicina62061016

**Published:** 2026-05-23

**Authors:** Luke Wojtalik, Thomas J. Sorenson, Amitesh Verma, Nolan Karp, Richard Shapiro

**Affiliations:** 1 Department of Surgery, NYU-Langone Health, New York, NY 10016, USA; 2 Hansjorg Wyss Department of Plastic Surgery, NYU-Langone Health, New York, NY 10016, USA; thomas.sorenson@nyulangone.org (T.J.S.);; 3 Grossman School of Medicine, NYU-Langone Health, New York, NY 10016, USA; amitesh.verma@nyulangone.org

**Keywords:** breast conserving therapy, breast reconstruction, mastectomy, breast cancer, breast implants

## Abstract

Breast cancer surgical management encompasses a spectrum of options that extend beyond oncologic control and carry substantially different cumulative surgical burdens. Although breast-conserving therapy (BCT) and mastectomy offer equivalent survival outcomes in many clinical scenarios, the downstream implications of these choices, including the number of operations, complication profiles, recovery timelines, and need for revision, are often underrecognized during initial treatment planning. This review aims to provide non-plastic surgeons with a practical framework for understanding the surgical burden associated with BCT compared with mastectomy and, when mastectomy is selected, the implications of subsequent reconstructive pathways. By discussing breast cancer surgery through the lens of cumulative surgical burden rather than isolated procedural choices, this review seeks to support more informed, multidisciplinary counseling and shared decision-making. A clearer understanding of reconstructive trajectories may help align surgical recommendations with patient values, optimize expectations, and reduce unanticipated downstream interventions across the continuum of breast cancer care.

## 1. Introduction

The surgical management of breast cancer has evolved significantly over the past several decades, shifting from radical extirpative approaches to more tailored, patient-centered strategies. Landmark trials established the oncologic equivalence of breast-conserving therapy (BCT) and mastectomy in appropriately selected patients, fundamentally altering treatment paradigms [1,2,3].

Despite this evolution, surgical decision-making often remains anchored in oncologic endpoints, with less emphasis placed on the cumulative surgical burden associated with each pathway. Surgical burden encompasses not only the index operation but also reoperations, complications, reconstructive procedures, recovery time, and long-term maintenance [4]. This gap is particularly relevant given that many patients undergo multiple procedures over the course of treatment, especially when reconstruction is involved [5,6]. Early decisions, such as choosing BCT versus mastectomy, set patients on trajectories with markedly different downstream implications. This review reframes breast cancer surgery through the lens of cumulative surgical burden, with the goal of equipping non-plastic surgeons with a clearer understanding of reconstructive pathways and their implications.

Importantly, the surgical burden associated with any given treatment pathway is highly context dependent and influenced by factors including tumor biology and stage, axillary management, radiation exposure, patient comorbidities, reconstructive technique, surgeon experience, and evolving practice patterns. Accordingly, the goal of this review is not to define fixed expectations for individual procedures, but rather to describe broad patterns and reconstructive trajectories that may inform individualized multidisciplinary counseling. Notably, surgical burden extends beyond operative interventions themselves and includes the physical, psychosocial, cognitive, and quality-of-life consequences experienced throughout treatment and survivorship.

This manuscript was designed as a narrative review and conceptual perspective intended to provide a practical framework for understanding cumulative surgical burden across breast cancer surgical pathways. A selective review of the literature was performed using PubMed and major breast surgery and reconstructive surgery literature. Emphasis was placed on landmark oncologic trials, contemporary reconstructive studies, patient-reported outcomes literature, and publications addressing reoperation rates, complications, reconstructive revisions, radiation-associated outcomes, and long-term maintenance considerations. The goal of this review was not to provide an exhaustive systematic synthesis, but rather to contextualize existing evidence within a clinically applicable framework for multidisciplinary counseling and shared decision-making.

## 2. Breast-Conserving Therapy Versus Mastectomy: Surgical Burden Comparison

Breast-conserving therapy (BCT) and mastectomy are oncologically equivalent for many patients with early-stage breast cancer [1,2,3]. However, equivalence in survival does not translate to equivalence in surgical experience. Each approach initiates a distinct treatment trajectory with different implications for the number of procedures, recovery burden, complication risk, and long-term maintenance (Table 1).

### 2.1. Number of Operations and Reintervention

BCT is often perceived as a single, less invasive procedure. In practice, however, it carries a meaningful likelihood of reoperation [7]. Historically, re-excision for positive or close margins has occurred in approximately 15–25% of patients undergoing BCT, although reported rates vary substantially based on tumor characteristics, institutional practice patterns, margin assessment approaches, and treatment era8. Importantly, adoption of consensus guidelines such as the Society of Surgical Oncology-American Society for Radiation Oncology (SSO-ASTRO) margin recommendations has contributed to declining re-excision rates in many contemporary practice settings [8].

In contrast, mastectomy typically achieves definitive oncologic resection in a single operation, with low rates of re-excision for margin control [9]. However, when reconstruction is pursued, the total number of operations often exceeds that of BCT [10]. Implant-based reconstruction commonly involves at least two stages (tissue expander placement followed by implant exchange), with additional revision procedures not uncommon [11]. Autologous reconstruction, while often completed in a single index operation, is also associated with secondary procedures for contouring, fat grafting, or symmetry [12]. As a result, the perceived simplicity of BCT versus mastectomy is misleading; BCT concentrates potential reinterventions early, whereas mastectomy, particularly with reconstruction, distributes surgical burden over time.

Importantly, these trajectories are highly variable and should not be interpreted as universally applicable. Some patients undergoing mastectomy without reconstruction may experience a relatively limited procedural course, whereas others pursuing complex reconstruction may undergo multiple staged or revision procedures over many years. Conversely, although BCT is often associated with lower procedural intensity, some patients ultimately require corrective surgery for asymmetry, fibrosis, contour deformity, or radiation-related changes. Thus, substantial overlap exists between these pathways, and cumulative surgical burden is shaped not only by the initial operation, but also by individual patient factors, adjuvant therapies, and evolving reconstructive needs over time.

### 2.2. Oncoplastic Surgical Techniques

An important contemporary consideration within breast-conserving therapy is the increasing use of oncoplastic surgical techniques. By combining oncologic resection with local tissue rearrangement or reduction-pattern reconstruction, oncoplastic surgery may expand eligibility for breast conservation in patients who historically may have required mastectomy due to unfavorable tumor-to-breast size ratios or anticipated contour deformity. In selected patients, these approaches may improve aesthetic outcomes and potentially reduce the likelihood of significant postoperative deformity or secondary corrective procedures [13,14].

At the same time, oncoplastic surgery introduces additional complexity that may alter the overall surgical burden profile of BCT. Bilateral symmetry procedures, larger tissue rearrangements, delayed wound healing, or revision procedures may still occur, particularly in the setting of radiation therapy [15,16,17]. Accordingly, oncoplastic surgery further illustrates that breast conservation and mastectomy exist along overlapping reconstructive and procedural spectra rather than as strictly separate categories of surgical intensity.

### 2.3. Recovery and Treatment Timeline

The recovery trajectory for BCT and mastectomy differs not only in duration but also in structure. BCT is typically associated with a shorter initial postoperative recovery, often allowing return to baseline activities within weeks [18]. However, this is followed by adjuvant radiation therapy, which typically requires daily treatments over 3–6 weeks [2]. While radiation is not a surgical procedure, it contributes meaningfully to overall treatment burden, including fatigue, skin toxicity, and disruption to daily life [19].

Mastectomy involves a more substantial upfront recovery, particularly when combined with reconstruction. Patients undergoing implant-based reconstruction may experience a staged recovery process, with expansion visits and subsequent procedures extending the overall timeline [20]. Autologous reconstruction entails the longest initial recovery, often including several days of hospitalization and a prolonged return to baseline function due to both chest and donor-site healing [21]. Thus, BCT is characterized by a shorter initial recovery but prolonged adjuvant treatment, whereas mastectomy, especially with reconstruction, front-loads surgical recovery but may reduce the need for radiation in select patients.

### 2.4. Complication Profiles

The complication profiles of BCT and mastectomy differ in both type and timing. BCT is generally associated with lower rates of acute surgical complications compared to mastectomy [22]. However, radiation therapy introduces a distinct set of delayed effects, including fibrosis, skin changes, breast asymmetry, and volume loss, which may evolve over time and occasionally require corrective procedures [23].

Mastectomy, particularly with reconstruction, carries higher rates of early surgical complications, including infection, seroma, wound healing issues, and, in the case of implants, device loss [24]. The risk profile is further modified by adjuvant radiation, which significantly increases complications in implant-based reconstruction, including capsular contracture and reconstructive failure [25].

Autologous reconstruction introduces additional risks related to microsurgery and donor-site morbidity, including flap loss (generally low but clinically significant), abdominal wall complications, and longer operative times [26]. Importantly, complications in the mastectomy pathway are more likely to result in additional operative interventions, whereas complications in BCT are more often managed nonoperatively but may contribute to long-term morbidity [27].

### 2.5. Radiation and Its Downstream Effects

Radiation plays a central role in differentiating these treatment pathways. BCT is traditionally associated with adjuvant radiation therapy, which remains a standard component of treatment for most patients [2]. However, contemporary de-escalation studies have demonstrated that omission of radiation may be appropriate in carefully selected low-risk populations, particularly older patients with favorable tumor biology and endocrine-responsive disease. As a result, the role and intensity of adjuvant radiation after BCT are becoming increasingly individualized in selected clinical settings.

In mastectomy patients, radiation is selectively applied based on tumor and nodal characteristics [28]. The presence or likelihood of radiation has profound implications for reconstruction. Implant-based reconstruction in the setting of radiation is associated with significantly higher rates of complications and failure, often necessitating additional surgeries or conversion to autologous reconstruction [29,30,31]. Conversely, autologous reconstruction is generally more resilient to radiation effects but may still be affected in terms of aesthetic outcomes [32]. Failure to account for anticipated radiation at the time of initial surgical planning can substantially increase downstream surgical burden.

### 2.6. Long-Term Surveillance and Maintenance

Patients undergoing BCT require ongoing breast imaging, typically with annual mammography, and may undergo additional imaging or biopsies for suspicious findings [33]. This surveillance introduces the potential for repeated diagnostic interventions and associated anxiety. After mastectomy, routine imaging of the reconstructed breast is generally not required, although clinical surveillance continues. However, reconstruction introduces its own form of long-term maintenance. Implant-based reconstruction may require future implant exchange, management of capsular contracture, or revision for aesthetic concerns [34]. Autologous reconstruction, while more durable, often involves secondary refinement procedures [12]. Thus, BCT is associated with ongoing diagnostic surveillance burden, whereas mastectomy, particularly with reconstruction, shifts burden toward procedural maintenance.

### 2.7. Patient-Reported Outcomes and Functional Considerations

Importantly, cumulative surgical burden is not solely procedural. Increasingly, patient-reported outcome studies demonstrate that the lived experience of breast cancer treatment encompasses physical, psychosocial, and emotional domains that may persist long after completion of surgery [35]. Measures such as BREAST-Q have shown meaningful differences across reconstructive pathways in satisfaction with breasts, psychosocial well-being, physical well-being, and sexual well-being [16,23]. While autologous reconstruction is often associated with higher long-term satisfaction and psychosocial outcomes in some studies, it may also involve greater short-term physical recovery and donor-site morbidity [36]. Implant-based reconstruction may offer shorter early recovery for selected patients but can be associated with anxiety related to complications, implant maintenance, or reconstructive failure. Similarly, patients undergoing BCT or aesthetic flat closure may experience highly variable outcomes related to body image, symmetry, radiation-associated changes, or satisfaction with contour and appearance [37,38]. Importantly, dissatisfaction and decisional regret are often linked less to the specific operation performed than to mismatch between expectations and the eventual longitudinal treatment experience [39]. As a result, understanding patient priorities and communicating likely trajectories of recovery, maintenance, and revision are critical components of patient-centered care.

### 2.8. Reframing the Comparison

A binary comparison between BCT and mastectomy obscures the more clinically relevant distinction: each option represents a different longitudinal surgical pathway. BCT concentrates interventions in the early postoperative and adjuvant period, with relatively lower procedural intensity but ongoing surveillance [7]. Mastectomy, particularly with reconstruction, often entails a higher upfront surgical burden with additional staged or revision procedures over time [10]. Recognizing these patterns is essential for counseling patients effectively. Early framing of treatment choices in terms of cumulative surgical burden (rather than isolated procedures) can better align surgical planning with patient priorities and reduce the likelihood of unanticipated downstream interventions.

Importantly, these patterns should not be interpreted as fixed characteristics of any individual pathway. Reoperation rates, complication profiles, revision burden, and long-term durability vary substantially based on patient selection, oncologic treatment requirements, reconstructive technique, institutional expertise, and contemporary practice patterns. As reconstructive strategies and perioperative management continue to evolve, the cumulative burden associated with each pathway may also shift over time.

## 3. Axillary Management as a Component of Surgical Burden

The cumulative burden of breast cancer surgery extends beyond management of the breast itself and is substantially influenced by the extent of axillary intervention. Although advances in surgical de-escalation have reduced the frequency of extensive axillary surgery in selected patients, axillary management remains a major determinant of postoperative morbidity, functional recovery, and long-term quality of life.

Sentinel lymph node biopsy (SLNB) is associated with substantially lower morbidity compared to axillary lymph node dissection (ALND) and has become standard for axillary staging in many clinically node-negative patients. In contrast, ALND carries significantly higher risks of lymphedema, shoulder dysfunction, pain, sensory disturbance, restricted range of motion, and prolonged postoperative recovery. These complications may persist long after completion of oncologic treatment and contribute meaningfully to physical and psychosocial burden throughout survivorship. Importantly, axillary morbidity may occur regardless of whether patients undergo breast conservation or mastectomy, further illustrating that cumulative surgical burden is shaped by the overall treatment pathway rather than the breast operation alone. In addition, adjuvant radiation involving the regional lymphatics may further increase risks of lymphedema and upper extremity dysfunction.

Contemporary efforts toward axillary de-escalation, including omission of ALND in selected patients with limited nodal disease, reflect a broader shift toward minimizing treatment-related morbidity while preserving oncologic safety. These evolving strategies align closely with the conceptual framework of cumulative surgical burden by recognizing that long-term functional outcomes and survivorship considerations are central components of breast cancer care.

## 4. Mastectomy and the Spectrum of Reconstruction

Selection of mastectomy represents a major inflection point in the surgical management of breast cancer, but it is not a singular decision. Rather, mastectomy initiates a series of downstream choices that define a patient’s reconstructive trajectory and, ultimately, their cumulative surgical burden. Framing reconstruction as a binary choice-reconstruction versus no reconstruction-fails to capture the diversity of available options and their distinct implications.

Instead, reconstruction after mastectomy should be understood as a spectrum of pathways, broadly categorized into: (1) aesthetic flat closure, (2) implant-based reconstruction, and (3) autologous reconstruction. Each pathway is associated with a unique profile of operative intensity, complication risk, need for revision, and long-term maintenance.

### 4.1. Divergent Surgical Trajectories

These reconstructive options differ not only in technique but in how surgical burden is distributed over time. Aesthetic flat closure (AFC) generally involves a single-stage procedure performed at the time of mastectomy, with relatively low long-term maintenance [40]. However, revision procedures for contour irregularities are not uncommon. Implant-based reconstruction often follows a staged approach, particularly when tissue expanders are used, resulting in multiple planned operations [11]. In addition, implant-based pathways are associated with a significant likelihood of unplanned revisions over time due to complications or device-related issues. [34]

Autologous reconstruction typically involves the highest upfront surgical burden, including longer operative times and recovery, but may offer greater long-term durability with fewer device-related complications [41]. Secondary procedures are common but are often elective refinements rather than responses to failure [12]. Understanding these trajectories is critical, as patients are not simply choosing a reconstructive modality-they are selecting a pattern of surgical care that may extend over months to years.

### 4.2. Interdependence of Early Decisions

Importantly, reconstructive pathways are not entirely interchangeable once treatment has begun. Early decisions regarding mastectomy type, timing of reconstruction, and anticipated adjuvant therapies can significantly influence the feasibility and outcomes of subsequent options [42]. For example, skin-sparing and nipple-sparing mastectomy techniques may facilitate improved aesthetic outcomes with both implant-based and autologous reconstruction but may not be oncologically appropriate in all patients [43]. These interdependencies underscore the importance of early, coordinated decision-making and multidisciplinary involvement.

### 4.3. Variability in Patient Priorities

The optimal reconstructive pathway is highly dependent on individual patient values, which may include: desire to minimize the total number of operations, preference for shorter initial recovery versus long-term durability, tolerance for implant maintenance or device-related risk, willingness to accept donor-site morbidity, and importance of breast mound restoration versus a flat contour [44,45]. No single pathway is universally “less burdensome.” Instead, each represents a tradeoff between upfront intensity and longitudinal maintenance, as well as between predictability and flexibility.

### 4.4. Common Misconceptions

Several misconceptions frequently arise during counseling:

• Reconstruction is optional, but not neutral: Choosing no reconstruction (AFC) still involves surgical decision-making and has aesthetic and functional consequences [40].
• Implants are not a one-time solution: Many patients will require additional procedures over time, even in the absence of complications [5].
• Autologous reconstruction is not a single surgery: Although often framed as definitive, it commonly includes staged revisions [46].
• All reconstructive pathways are equally compatible with radiation: In reality, radiation disproportionately affects implant-based outcomes [29].

Failure to address these misconceptions can lead to misaligned expectations and dissatisfaction.

### 4.5. Implications for Surgical Counseling

For non-plastic surgeons, the key implication is that the decision to proceed with mastectomy should prompt early and explicit discussion of reconstructive pathways, rather than deferring these considerations until after oncologic planning is complete [47]. At a minimum, patients should understand that:

• Mastectomy does not eliminate the possibility of multiple future procedures
• Reconstruction type significantly influences complication risk and long-term course
• Anticipated adjuvant therapies, particularly radiation, may alter the optimal pathway

Early referral to plastic surgery is essential to ensure that patients are counseled on the full spectrum of options and their associated trajectories before committing to a surgical plan.

## 5. Aesthetic Flat Closure

Aesthetic flat closure (AFC) is an increasingly recognized reconstructive pathway following mastectomy, yet it remains frequently misunderstood in both clinical practice and patient counseling. Often described as “no reconstruction,” AFC is more accurately characterized as an intentional reconstructive approach aimed at achieving a smooth, symmetric, and contour-optimized chest wall.

### 5.1. Defining Aesthetic Flat Closure

AFC extends beyond simple mastectomy skin closure. It involves deliberate surgical planning to: eliminate excess skin and soft tissue redundancy, address lateral chest wall fullness and “dog ears”, and create a symmetric and aesthetically acceptable flat contour [48]. In patients with larger or ptotic breasts, this may require significant soft tissue rearrangement and contouring [49]. As such, AFC should be viewed as an active reconstructive procedure, not the absence of one.

### 5.2. Surgical Technique and Considerations

The technical execution of AFC varies based on patient anatomy and oncologic constraints. Key considerations include:

• Skin envelope management: Determining the appropriate amount of skin excision to avoid both redundancy and excessive tension [49]
• Lateral contouring: Addressing axillary and lateral chest wall fullness, which is a common source of postoperative dissatisfaction [49,50]
• Symmetry: Particularly relevant in bilateral procedures, but also in unilateral cases where contralateral procedures may be considered [51]

Failure to address these elements at the index operation can result in contour deformities that are difficult to correct secondarily.

### 5.3. Complications and Revision Burden

AFC is often perceived as the lowest-risk, lowest-burden pathway. While it avoids implant- and flap-related complications, it is not without morbidity. Common postoperative issues include seroma formation, wound healing complications, and residual contour irregularities [52]. Importantly, revision procedures are not uncommon, particularly for correction of lateral fullness, redundant tissue, or asymmetry [40]. These revisions may range from minor outpatient procedures to more involved surgical corrections. Although the overall complication profile is generally favorable compared to reconstructive alternatives, the assumption that AFC represents a “one-and-done” approach is not consistently accurate.

### 5.4. Patient Selection and Counseling

Patients choosing AFC often prioritize minimizing the number of surgeries, avoiding implants or donor-site morbidity, and shorter recovery and lower procedural intensity [53,54]. However, these preferences must be balanced with a clear understanding of expected outcomes. Key counseling points include:

• The aesthetic result is a flat contour, not a restoration of breast volume
• Residual asymmetry or contour irregularities may occur
• Secondary revisions may be required to optimize appearance

Patients with higher body mass index, significant lateral tissue excess, or marked preoperative asymmetry may have a higher likelihood of requiring revision procedures [53].

### 5.5. Common Misconceptions and Sources of Dissatisfaction

AFC is particularly vulnerable to miscommunication and unmet expectations. Common misconceptions include equating AFC with a simple closure rather than a reconstructive procedure, underestimating the importance of surgical technique in achieving a satisfactory contour, and if declining reconstruction eliminates the possibility of future procedures [40,49,55]. Importantly, dissatisfaction following AFC is often driven not only by technical outcomes, but also by communication failures and mismatched expectations [51]. Increasing attention has been directed toward the phenomenon of “flat denial,” in which patients desiring a flat result perceive that the option was inadequately presented, discouraged, or incompletely executed despite their stated preferences [37,56]. Historically, AFC has not always been consistently framed as a valid reconstructive pathway equivalent to implant-based or autologous reconstruction, contributing to variability in counseling quality and patient expectations [40].

As a result, patient satisfaction after AFC appears to depend heavily on clear preoperative communication regarding contour expectations, lateral chest wall appearance, potential need for revision procedures, and the intentional reconstructive goals of the operation. These considerations reinforce the broader principle that cumulative surgical burden is shaped not only by procedural complexity, but also by the quality of shared decision-making and alignment between patient goals and the eventual treatment trajectory.

### 5.6. Position Within the Surgical Burden Framework

Within the broader context of surgical burden, AFC generally represents a pathway with lower upfront operative complexity compared to autologous reconstruction, fewer planned staged procedures compared to implant-based reconstruction, and limited long-term maintenance requirements. However, this lower overall burden is contingent on appropriate patient selection, meticulous surgical technique, and realistic expectation setting. When these factors are not aligned, the need for revision procedures can erode the perceived simplicity of this pathway. AFC should therefore be presented not as the absence of reconstruction, but as a distinct reconstructive strategy with its own tradeoffs, risks, and potential need for refinement.

## 6. Implant-Based Breast Reconstruction (IBBR)

Implant-based breast reconstruction remains the most utilized reconstructive approach following mastectomy, largely due to its relative technical accessibility, shorter initial operative time compared to autologous reconstruction, and avoidance of donor-site morbidity [57]. However, despite its perceived simplicity, implant-based reconstruction is associated with a distinct and often underappreciated longitudinal surgical burden, characterized by staged procedures, susceptibility to complications, and ongoing maintenance over time [58].

### 6.1. Reconstructive Approaches: Staged vs. Direct-to-Implant

Implant-based reconstruction is typically performed using one of two strategies [59]:

• Two-stage reconstruction, involving placement of a tissue expander at the time of mastectomy followed by a second operation for exchange to a permanent implant
• Direct-to-implant (DTI) reconstruction, in which a permanent implant is placed at the index operation in carefully selected patients

While DTI offers the potential to reduce the number of planned operations, it is not universally applicable. Patient selection depends on factors such as skin flap quality, breast size, comorbidities, and anticipated adjuvant therapies [60,61]. Even in DTI cases, secondary procedures for revision or optimization remain common [62].

### 6.2. Role of Mesh and Soft Tissue Support

Acellular dermal matrix (ADM) and, more recently, synthetic meshes are frequently used to support implant positioning and define the implant pocket [63,64,65]. These materials have facilitated broader adoption of prepectoral reconstruction and DTI approaches [66,67]. However, their use introduces additional considerations: increased material cost, potential contribution to seroma or infection, and variability in integration and long-term outcomes [68]. While widely accepted, mesh-assisted reconstruction is not without tradeoffs and does not eliminate the need for revision procedures [69].

### 6.3. Complication Profile

Implant-based reconstruction is associated with a range of early and late complications, including infection and cellulitis, seroma and hematoma, skin flap necrosis, and implant exposure or loss [58]. Reported rates of reconstructive failure (implant loss) vary but are generally in the range of 5–15%, depending on patient factors and treatment context [70,71,72]. Even in the absence of failure, complications frequently necessitate additional interventions due to the presence of a foreign implant [34].

### 6.4. Impact of Radiation Therapy

Radiation is one of the most significant modifiers of outcomes in implant-based reconstruction. Patients undergoing post-mastectomy radiation therapy experience higher rates of capsular contracture, increased risk of infection and wound complications, and greater likelihood of implant loss [25,73]. In radiated fields, reconstructive failure rates may exceed 20–30% in some series, and aesthetic outcomes are often compromised [74,75]. As a result, patients initially planned for implant-based reconstruction may ultimately require conversion to autologous reconstruction, increasing overall surgical burden [30]. Failure to account for the likelihood of radiation at the time of initial reconstruction planning is a major contributor to unanticipated downstream procedures.

### 6.5. Revision Procedures and Long-Term Maintenance

A defining feature of implant-based reconstruction is the high likelihood of revision over time. Even in uncomplicated cases, patients frequently undergo additional procedures for implant malposition or asymmetry, capsular contracture, rippling or contour deformities, or size adjustment or aesthetic refinement [34,76,77]. Estimates suggest that a substantial proportion of patients-often 30–50% or more-undergo at least one revision procedure within several years of reconstruction [5,34,62]. In addition, implants are not lifetime devices. Many patients will require implant exchange at some point due to device aging, complications, or evolving aesthetic preferences [78].

### 6.6. Patient Selection and Tradeoffs

Implant-based reconstruction is often favored by patients seeking shorter initial operative time, avoidance of donor-site morbidity, and faster early recovery compared to autologous reconstruction [44]. However, these advantages must be balanced against a higher likelihood of staged procedures, increased sensitivity to radiation and ongoing risk of complications and revision. For some patients, implant-based reconstruction represents a lower upfront burden but a higher cumulative burden over time.

### 6.7. Position Within the Surgical Burden Framework

Within the broader framework of surgical burden, implant-based reconstruction is best understood as a moderate upfront, high longitudinal burden pathway. While the initial operation may be shorter and less physiologically demanding than autologous reconstruction, the need for staged procedures, revisions, and long-term maintenance often results in a greater total number of interventions over the patient’s lifetime. This distinction is critical for patient counseling. Presenting implant-based reconstruction as a “simpler” option without acknowledging its long-term trajectory risks misalignment between expectations and experience.

Importantly, substantial variability exists within implant-based reconstruction pathways. Some patients experience relatively uncomplicated courses with limited long-term intervention requirements, while others undergo multiple revisions related to capsular contracture, implant malposition, infection, or reconstructive failure. This variability reflects differences in patient selection, radiation exposure, reconstructive technique, and longitudinal follow-up.

## 7. Autologous Breast Reconstruction

Autologous breast reconstruction utilizes the patient’s own tissue to recreate a breast mound, most commonly through abdominally based free flaps such as the deep inferior epigastric perforator (DIEP) flap [79]. This approach represents a fundamentally different reconstructive philosophy compared to implant-based reconstruction, emphasizing biological tissue replacement rather than prosthetic substitution. Within the surgical burden framework, autologous reconstruction is characterized by a high upfront operative investment with comparatively lower long-term maintenance, though secondary procedures remain common.

### 7.1. Operative Complexity and Initial Recovery

Autologous reconstruction is among the most technically demanding procedures in breast surgery. Free flap reconstruction typically involves prolonged operative times, often ranging from 6 to 10+ h, microsurgical anastomosis requiring specialized expertise, and multidisciplinary perioperative care [80]. Hospital length of stay is correspondingly longer, commonly 3–5 days, with extended recovery due to healing at both the chest and donor site [81]. Patients must also tolerate a more substantial immediate postoperative course, including activity restrictions and a longer timeline to return to baseline function compared to implant-based reconstruction [82].

### 7.2. Donor-Site Morbidity

A defining feature of autologous reconstruction is the presence of donor-site morbidity. In abdominally based flaps, this may include abdominal wall weakness or bulge, hernia formation, and wound healing complications [83]. While muscle-sparing techniques such as the DIEP flap reduce these risks compared to earlier methods, donor-site effects remain an inherent tradeoff. For some patients, the abdominal contour change is perceived as a benefit; for others, it represents a meaningful source of morbidity [84].

### 7.3. Complication Profile

Autologous reconstruction carries risks associated with both microsurgery and general surgical morbidity. Key complications include total or partial flap loss (typically ~1–5% for total loss in experienced centers), vascular compromise requiring urgent reoperation, fat necrosis, which may present as palpable masses or contour irregularities, and wound complications at the breast or donor site [85,86,87]. Importantly, while flap loss is relatively uncommon, it is a high-impact complication that often necessitates additional major surgery [88,89]. Compared to implant-based reconstruction, complications in autologous reconstruction are more likely to occur early and be managed definitively, rather than recurring over time.

### 7.4. Durability and Long-Term Outcomes

One of the principal advantages of autologous reconstruction is its long-term durability. Because the reconstructed breast consists of living tissue, there is no risk of device-related failure. However, the reconstructed breast may change with the patient’s weight and aging; though rates of long-term structural complications are generally lower than with implants [90]. Autologous reconstruction is also more resilient in the setting of radiation [70]. Although radiation can still impact aesthetic outcomes, it is less likely to result in catastrophic reconstructive failure compared to implant-based approaches [91].

### 7.5. Revision Procedures

Despite its reputation as a “definitive” reconstruction, autologous reconstruction frequently involves secondary procedures. These are typically performed to optimize aesthetic outcomes and may include fat grafting to improve contour or volume, revision of the breast mound or inframammary fold, and symmetrizing procedures on the contralateral breast [12]. Unlike implant-related revisions, these procedures are often elective refinements rather than responses to complications or device failure [5]. However, they contribute meaningfully to the total number of operations a patient undergoes.

### 7.6. Patient Selection and Tradeoffs

Autologous reconstruction is often favored by patients who desire a more natural feel and appearance, prefer to avoid implants and long-term device maintenance, are undergoing or likely to require radiation therapy, and are willing to accept a longer initial recovery for improved long-term stability [92]. However, it may be less suitable for patients with significant comorbidities, limited donor tissue, or those who prioritize shorter operative time and faster early recovery [93].

### 7.7. Position Within the Surgical Burden Framework

Within the context of surgical burden, autologous reconstruction is best understood as a high upfront, lower long-term burden pathway. The initial operation is longer, more complex, and associated with a more demanding recovery period. However, this is often offset by lower rates of long-term reconstructive failure, reduced need for device-related maintenance, and greater stability of outcomes over time. That said, the total surgical burden remains substantial, particularly when accounting for secondary refinement procedures. The advantage of this pathway lies less in minimizing the number of operations and more in shifting surgical burden toward a defined early period, rather than distributing it unpredictably over the long term.

## 8. Reconstruction Timing: Delayed Versus Immediate

The timing of breast reconstruction, i.e., whether performed at the time of mastectomy (immediate) or at a later stage following completion of oncologic therapy (delayed), is a critical determinant of cumulative surgical burden. While often framed as a logistical or patient-preference decision, timing has substantial implications for complication risk, reconstructive success, and the total number of procedures required over the course of treatment.

### 8.1. Immediate Reconstruction

Immediate reconstruction is performed concurrently with mastectomy and has become increasingly common due to its aesthetic and psychological advantages. Potential advantages include preservation of the native skin envelope, often enabling improved aesthetic outcomes, reduction in the total number of operative settings in selected cases, and avoidance of a period without a breast mound, which may be important for patient well-being [94]. However, these benefits must be weighed against important limitations.

Immediate reconstruction requires patients to make complex reconstructive decisions at the time of cancer diagnosis, often under significant emotional stress [95]. In addition, reconstructive planning must occur in the context of incomplete oncologic information, particularly with respect to the need for adjuvant radiation [96].

When radiation is delivered following immediate reconstruction, especially in implant-based pathways, it is associated with increased complication rates, higher risk of reconstructive failure, and inferior aesthetic outcomes [97,98]. As a result, patients undergoing immediate reconstruction in the setting of unanticipated radiation may experience a higher cumulative surgical burden, including revision procedures or conversion to alternative reconstructive strategies.

### 8.2. Delayed Reconstruction

Delayed reconstruction is performed after completion of oncologic treatment, including chemotherapy and radiation when indicated. Advantages of delayed reconstruction include an ability to incorporate definitive oncologic information, particularly radiation status, into reconstructive planning, potential reduction in complication rates, especially in implant-based reconstruction, and greater flexibility in selecting the most appropriate reconstructive modality [99].

Delayed reconstruction may be particularly advantageous in patients with a high likelihood of requiring post-mastectomy radiation therapy, as it allows reconstruction to be tailored to a radiated field rather than exposing a newly reconstructed breast to radiation-related complications [96]. However, delayed reconstruction introduces its own challenges, including requiring an additional major operation after completion of cancer treatment, prolonging the overall treatment timeline, possibly resulting in less favorable aesthetic outcomes due to loss of the native skin envelope, and requiring patients to undergo a period without breast reconstruction, which may be psychologically distressing [100].

Importantly, contemporary reconstructive practice increasingly incorporates intermediate and individualized timing strategies that extend beyond a strict immediate-versus-delayed framework. Delayed-immediate approaches, in which temporary tissue preservation strategies are used pending final pathologic or radiation decisions, may help balance aesthetic preservation with oncologic flexibility [99]. Similarly, advances in prepectoral implant-based reconstruction, improved mastectomy flap assessment, and evolving multidisciplinary risk stratification have enabled more tailored reconstructive sequencing based on estimated likelihood of post-mastectomy radiation therapy [29,101]. As a result, reconstruction timing is increasingly determined through individualized assessment of tumor biology, anticipated adjuvant therapy, patient priorities, and reconstructive goals rather than through fixed categorical pathways alone.

### 8.3. Decisional Timing and Patient Burden

Timing also influences the cognitive and emotional burden placed on patients. Immediate reconstruction requires patients to process complex information and make high-stakes decisions at the time of diagnosis [102]. Delayed reconstruction allows for more time and deliberation but extends the overall treatment course. This tradeoff between decisional urgency and procedural staging should be explicitly addressed during counseling.

### 8.4. Position Within the Surgical Burden Framework

Within the framework of surgical burden, timing determines not only how many procedures a patient undergoes, but also when and under what conditions those procedures occur. Immediate reconstruction may reduce the number of discrete operative events but can increase complication-driven interventions if oncologic variables, particularly radiation, are not fully accounted for. Delayed reconstruction may increase the number of planned operations but can reduce unplanned procedures and improve reconstructive reliability in selected patients. No single timing strategy is universally optimal. Rather, the goal is to align timing with oncologic certainty, patient priorities, and reconstructive goals in a way that minimizes unanticipated downstream interventions.

## 9. Shared Decision Making and Patient Counseling

The following section builds upon the preceding literature review to propose a conceptual framework for integrating cumulative surgical burden into multidisciplinary counseling and shared decision-making. Given the complexity and variability of breast cancer surgical pathways, effective decision-making requires a structured, patient-centered approach that extends beyond selection of an index operation. Framing choices in terms of longitudinal surgical burden, rather than isolated procedures, can improve alignment between treatment plans and patient expectations (Table 2).

### 9.1. Reframing the Clinical Conversation

Traditional counseling often presents decisions sequentially: lumpectomy versus mastectomy, followed by reconstruction versus no reconstruction [103]. This stepwise approach can obscure the downstream implications of early choices. Instead, discussions should be reframed around anticipated treatment trajectories, emphasizing that each option represents a pathway with distinct patterns of number and timing of procedures, likelihood of unplanned interventions, recovery burden over time, and long-term maintenance requirements [104]. Explicitly communicating that there is no universally “simpler” option, and that tradeoffs are inherent to each pathway, helps establish realistic expectations from the outset.

### 9.2. Core Domains to Clarify Early

Several key factors should be addressed early in the decision-making process, as they significantly influence optimal pathway selection:

1. Likelihood of Radiation Therapy
  Anticipated radiation is one of the most important determinants of reconstructive success and complication risk. Early assessment, based on tumor characteristics and nodal status, should inform both reconstructive modality and timing.
2. Patient Priorities and Tolerance for Surgical Burden
  Patients vary in their willingness to undergo multiple procedures, accept prolonged recovery, or tolerate the possibility of revision surgery [44]. The following clarifying preferences regarding can guide selection of the most appropriate pathway:

- Upfront versus staged procedures
- Long-term maintenance
- Aesthetic goals

3. Medical and Anatomic Considerations
  Comorbidities, body habitus, prior surgeries, and smoking status may influence eligibility for specific reconstructive options and associated risks [105].

4. Oncologic Timeline and Urgency
  The need for timely cancer treatment may limit reconstructive options or favor staged approaches.

Failure to address these domains early can lead to suboptimal sequencing of care and increased downstream interventions.

### 9.3. Role and Timing of Plastic Surgery Consultation

Early involvement of plastic surgery is critical to comprehensive counseling. Referral should ideally occur prior to definitive surgical planning, rather than after a mastectomy decision has been finalized [106]. Delayed referral can result in limited reconstructive options, suboptimal timing relative to radiation, and increased likelihood of revision or conversion procedures. Multidisciplinary coordination allows for alignment of oncologic and reconstructive goals and facilitates more informed patient decision-making.

### 9.4. Communicating Uncertainty and Risk

A central challenge in counseling is the inherent uncertainty in predicting individual outcomes. Rather than presenting overly definitive recommendations, surgeons should communicate risk in terms of ranges and probabilities, including, likelihood of requiring additional operations, risk of complications and reconstructive failure, and impact of adjuvant therapies on outcomes [107]. Framing uncertainty transparently helps patients make decisions that are resilient to unexpected developments, such as the need for radiation or postoperative complications.

### 9.5. Practical Counseling Framework

A structured approach to counseling may include the following steps:

1. Define the Oncologic Context: Clarify eligibility for BCT versus mastectomy and assess likelihood of radiation.
2. Introduce Reconstruction as a Spectrum: Present aesthetic flat closure, implant-based reconstruction, and autologous reconstruction as distinct pathways with different trajectories.
3. Discuss Timing Options in Context: Integrate immediate versus delayed reconstruction into the broader discussion, particularly in relation to radiation risk.
4. Elicit Patient Values Explicitly: Ask targeted questions to understand preferences regarding recovery, number of procedures, and long-term outcomes.
5. Align Recommendations with Priorities: Tailor guidance based on the intersection of oncologic factors and patient values, rather than defaulting to a single reconstructive approach.

### 9.6. Position Within the Surgical Burden Framework

Shared decision-making serves as the mechanism through which the concept of surgical burden is operationalized in clinical practice. By explicitly incorporating cumulative burden into counseling, surgeons can reduce the likelihood of unanticipated downstream procedures, improve patient satisfaction and expectation alignment, and facilitate more efficient and coordinated care. Importantly, the goal is not to direct patients toward a particular pathway, but to ensure that their chosen approach reflects a clear understanding of the full trajectory of care, rather than the perceived simplicity of an individual procedure.

## 10. Future Directions

As breast cancer treatment continues to evolve, there is increasing recognition that surgical success should be defined not only by oncologic outcomes, but also by the cumulative burden imposed on patients over the course of care. The perspectives outlined below are intended not as prescriptive recommendations, but as potential directions through which evolving evidence and multidisciplinary care models may further reduce cumulative surgical burden and improve patient-centered decision-making.

Several emerging trends have the potential to reshape how surgical burden is understood and managed. First, there is a growing movement toward de-escalation of surgery in appropriately selected patients [108]. Advances in systemic therapy and tumor biology are enabling more tailored approaches to both breast and axillary surgery, with the goal of minimizing unnecessary interventions while maintaining oncologic safety [109]. As these strategies mature, they may reduce the number and extent of surgical procedures required across the treatment continuum.

An additional emerging area relevant to surgical burden is the effort to avoid unnecessary mastectomy in selected patients demonstrating exceptional response to neoadjuvant systemic therapy [110]. Increasingly effective systemic treatments, particularly in biologically responsive tumor subtypes, have expanded interest in surgical de-escalation strategies and the potential for breast conservation even in patients who historically may have undergone mastectomy. Advances in imaging, response assessment, and tumor biology are contributing to a more individualized approach in which extent of surgery may increasingly reflect treatment response rather than initial disease presentation alone. These evolving strategies further reinforce the importance of viewing breast cancer treatment longitudinally, with emphasis not only on oncologic safety but also on minimizing cumulative surgical and reconstructive burden where appropriate.

Next, the integration of patient-reported outcomes (PROs) is shifting the focus from procedure-centered metrics to patient-centered measures of success [111]. Traditional endpoints such as complication rates and operative time fail to fully capture the lived experience of treatment, including recovery burden, aesthetic satisfaction, and quality of life. Incorporating PROs into clinical decision-making and research will be essential for accurately evaluating the tradeoffs between reconstructive pathways.

Third, there is increasing emphasis on multidisciplinary and longitudinal counseling models. Early collaboration between surgical oncology, plastic surgery, radiation oncology, and medical oncology allows for more accurate prediction of treatment trajectories and better alignment of surgical planning with anticipated adjuvant therapies [112]. Structured counseling frameworks that explicitly address surgical burden may improve both decision quality and patient satisfaction [113].

Finally, advances in reconstructive techniques, including refinement of autologous flap surgery, evolving implant technologies, and hybrid approaches, may further expand available options [59,114,115]. However, innovation alone will not reduce surgical burden unless it is paired with thoughtful patient selection and transparent counseling regarding long-term implications. Collectively, these developments suggest a shift toward more individualized, trajectory-based care, in which treatment decisions are guided not only by oncologic factors, but also by a comprehensive understanding of their downstream consequences.

## 11. Conclusions

Breast cancer surgery is best understood not as a single decision, but as a sequence of interconnected choices that shape a patient’s experience over time. While breast-conserving therapy and mastectomy often provide equivalent oncologic outcomes, they initiate different treatment trajectories with distinct patterns of procedural intensity, recovery, revision burden, surveillance, and long-term maintenance. When mastectomy is selected, reconstructive options represent divergent pathways rather than interchangeable procedures, each associated with unique tradeoffs related to operative burden, complication risk, and longitudinal care. Recognizing these differences may help support more transparent counseling, multidisciplinary planning, and expectation setting. Framing treatment decisions through the lens of cumulative surgical burden offers a practical conceptual approach for contextualizing the downstream implications of surgical and reconstructive choices alongside traditional oncologic considerations. Although this framework requires further study and prospective validation, it may provide a useful patient-centered perspective for understanding the broader experience of breast cancer treatment and survivorship.

## Figures and Tables

**Table 1:** Comparison of Surgical Burden: Breast-Conserving Therapy vs. Mastectomy.

Domain	Breast-Conserving Therapy (BCT)	Mastectomy with Aesthetic Flat Closure (AFC)	Mastectomy with IBBR	Mastectomy with Immediate ABR	Mastectomy with Delayed ABR
Initial Operation	Lumpectomy	Mastectomy	Mastectomy + expander (“2-stage” or implant (“DTI”) placement	Mastectomy + free flap reconstruction	Mastectomy + expander placement
Number of Planned Operations	One minor	One major	- DTI: one major + possible minor - 2-stage: one major + guaranteed minor	One major + possible minor	Two major + possible minor
Reoperation/Revision Rates	- Possible re-excision - Possible conversion to mastectomy - Possible aesthetic revisions	Low, if performed correctly	Higher for refinement (fat grafting, symmetry)	Higher for refinement (fat grafting, symmetry)	Higher for refinement (fat grafting, symmetry)
Recovery (Initial)	Short (one week)	Moderate (few weeks)	Moderate (staged recovery)	Prolonged (weeks to months)	Prolonged (weeks to months)
Adjuvant Therapy Burden	Radiation required in most patients	Selective radiation	Radiation significantly increases complications	More tolerant of radiation (aesthetic impact possible)	More tolerant of radiation (aesthetic impact possible)
Complication Profile	- Lower acute surgical complications - Radiation-related fibrosis with asymmetry	Surgical complications (infection, wound, seroma)	- Surgical complications - Implant loss - Capsular contracture	- Surgical complications - Flap loss, fat necrosis - Donor-site morbidity	- Surgical complications - Expander loss - Flap loss, fat necrosis - Donor-site morbidity
Long-Term Maintenance	Ongoing imaging and possible biopsies	Minimal	- Device related maintenance - Possible revisions (implant exchange, fat grafting, nipple reconstruction)	- Possible revisions (fat grafting, mastopexy/skin excision, nipple reconstruction)	- Possible revisions (fat grafting, mastopexy/skin excision, nipple reconstruction)
Total Surgical Burden Pattern	Lower upfront surgery, ongoing surveillance and radiation burden	Lowest procedural burden overall	Moderate upfront, higher longitudinal procedural burden	Higher upfront burden, lower long-term procedural maintenance	Higher upfront burden, lower long-term procedural maintenance

KEY: IBBR implant-based breast reconstruction, ABR autologous breast reconstruction, DTI direct to implant.

**Table 2:** Patient Priorities and Corresponding Reconstructive Pathways.

Patient Priority/Clinical Scenario	Favored Pathway(s)	Key Advantages	Key Tradeoffs/Risks
Minimize total number of surgeries	AFC	- Single-stage approach if done correctly - Minimal long-term maintenance	- Potential for contour dissatisfaction
Avoid implants or foreign materials	AFC or ABR	- No implant-related complications or maintenance	- AFC: no breast mound - ABR: higher upfront burden, donor-site morbidity
Shortest surgery	IBBR (DTI or 2-stage)	- Faster early recovery - No donor site morbidity	- DTI: long-term maintenance of implants - 2-stage: at least two operations, long-term maintenance of implants
Minimize maintenance	AFC or ABR	- No implant-related complications or maintenance - Long-term natural feel	- AFC: no breast mound - ABR: higher upfront burden, donor-site morbidity
Strong preference to avoid period without breast mound	DTI or immediate ABR	- Maintains body image continuity	DTI: long-term maintenance of implants ABR: higher upfront burden, donor-site morbidity
Wants substantially larger reconstructed breasts	2-stage IBBR	- Size per patient preference	- At least two operations - Requires serial expansions - Longer time to reconstruction completion
Significant comorbidities/poor surgical candidate	AFC or simple IBBR	- Shortest surgery with lowest physiologic stress	- May limit reconstructive options or aesthetic outcomes
Desire for most natural feel/appearance	ABR	- More natural contour and feel - Ages with patient	- Higher upfront burden - May require revision if body habitus changes
Low BMI or unavailable abdomen donor site	IBBR or nonabdominal ABR	- Allows for breast reconstruction even with poor ABR donor sites	- IBBR: long term maintenance of implants - Nonabdominal ABR: requires more specialized surgeons, higher upfront burden, donor-site morbidity

Key: AFC aesthetic flat closure, ABR autologous breast reconstruction, DTI direct to implant, IBBR implant-based breast reconstruction.

## Data Availability

No new data were created or analyzed in this study.
